# Natural language processing for populating lung cancer clinical research data

**DOI:** 10.1186/s12911-019-0931-8

**Published:** 2019-12-05

**Authors:** Liwei Wang, Lei Luo, Yanshan Wang, Jason Wampfler, Ping Yang, Hongfang Liu

**Affiliations:** 1 Department of Health Sciences Research, Mayo Clinic College of Medicine, Rochester, MN 55901 USA; 2 Department of Good Clinical Practice, Guizhou Province People’s Hospital, Guiyang, China

**Keywords:** Natural language processing, Lung cancer, Stage, Histology, Tumor grade, Treatments

## Abstract

**Background:**

Lung cancer is the second most common cancer for men and women; the wide adoption of electronic health records (EHRs) offers a potential to accelerate cohort-related epidemiological studies using informatics approaches. Since manual extraction from large volumes of text materials is time consuming and labor intensive, some efforts have emerged to automatically extract information from text for lung cancer patients using natural language processing (NLP), an artificial intelligence technique.

**Methods:**

In this study, using an existing cohort of 2311 lung cancer patients with information about stage, histology, tumor grade, and therapies (chemotherapy, radiotherapy and surgery) manually ascertained, we developed and evaluated an NLP system to extract information on these variables automatically for the same patients from clinical narratives including clinical notes, pathology reports and surgery reports.

**Results:**

Evaluation showed promising results with the recalls for stage, histology, tumor grade, and therapies achieving 89, 98, 78, and 100% respectively and the precisions were 70, 88, 90, and 100% respectively.

**Conclusion:**

This study demonstrated the feasibility and accuracy of automatically extracting pre-defined information from clinical narratives for lung cancer research.

## Background

Lung cancer is the second most common cancer and by far the leading cause of cancer-related death in both men and women, accounting for 1 in 4 cancer deaths in U.S. [1]. Accurate identification of lung cancer related information is very important for epidemiological studies, especially in terms of prognosis [2, 3], which in turn is critical for improving cancer outcomes. There are two main types of lung cancer, non-small cell lung cancer (NSCLC) (80–85% of cases) and small cell lung cancer (SCLC) (15–20% of cases) [4]. Three major therapeutic options for lung cancer include surgery, regional radiation therapy, and systemic drug therapy [5]. Cancer stage and other factors, such as histology and tumor grade have been used by doctors to choose various treatment plans [6]. Stage and treatment modality have been the most important factors for lung cancer prognosis [3]. Different histological types of lung cancer are associated with different survival, e.g., highest survival in patients with bronchioloalveolar adenocarcinoma and lowest in those with small and large cell tumors [7]. Histological subtypes of NSCLC also provide important information for drug selection [4].

Epidemiologists use electronic health records (EHR) with rich longitudinal data on large populations for epidemiologic research [8]. Since manual review of large volumes of text materials is time consuming and labor intensive, some efforts have emerged to automatically extract information from text using natural language processing (NLP), an artificial intelligence technique.

Most information extraction systems that support at the point of care and enable secondary use of EHRs for clinical, epidemiological and translational research are expert-based systems [9]. Nguyen AN et al. employed symbolic rule-based approach using SNOMED CT to automatically extract lung cancer stages from free-text pathology reports based on the tumor, node, metastasis (TNM) stage [10]. The overall accuracy was 72, 78, and 94% for T, N, and M staging, respectively. Warner et al. automatically extracted overall stage of lung cancer from narrative texts in EHR [11] using exact stage (e.g., stage I and stage IV) and inexact stage (e.g., “early stage”), without indicating what the narrative text included. The stage accuracy was high compared with the gold standard with k = 0.906 (95% CI, 0.873 to 0.939). Zheng et al. used clinical notes to automatically extract chemotherapy and radiotherapy information in lung cancer patients with the Information and Data Extraction using Adaptive Learning (IDEAL-X) system [12]. The system achieved an overall precision of over 93%. A recent study used pathology reports to detect metastatic status (including histological type, tumor grade, specimen site, metastatic status indicators and the procedure) and metastasis site [13]. This system achieved a recall and precision of 0.84 and 0.88 for detecting metastatic status. DeepPhe enables automated extraction of cancer phenotype information including histological types and tumor stages from EHR, showing agreement with human expert extracted information ranged from 0.20 to 0.96 [14], but it does not include lung cancer.

In this study, we developed and evaluated an information extraction system to capture information on stage, histology, tumor grade and therapies in lung cancer patients using various clinical narrative documents including clinical notes, pathology reports and surgery reports. For therapies, we extracted chemotherapy (one of drug therapy), radiotherapy and surgery. We focused on only primary lung cancer, and therefore metastasis is not considered in the system. Contribution of our study lies in the capability of the NLP system to reduce labors of human abstractors and improve efficacy of data extraction for lung cancer clinical research.

## Methods

**Fig. 1:**
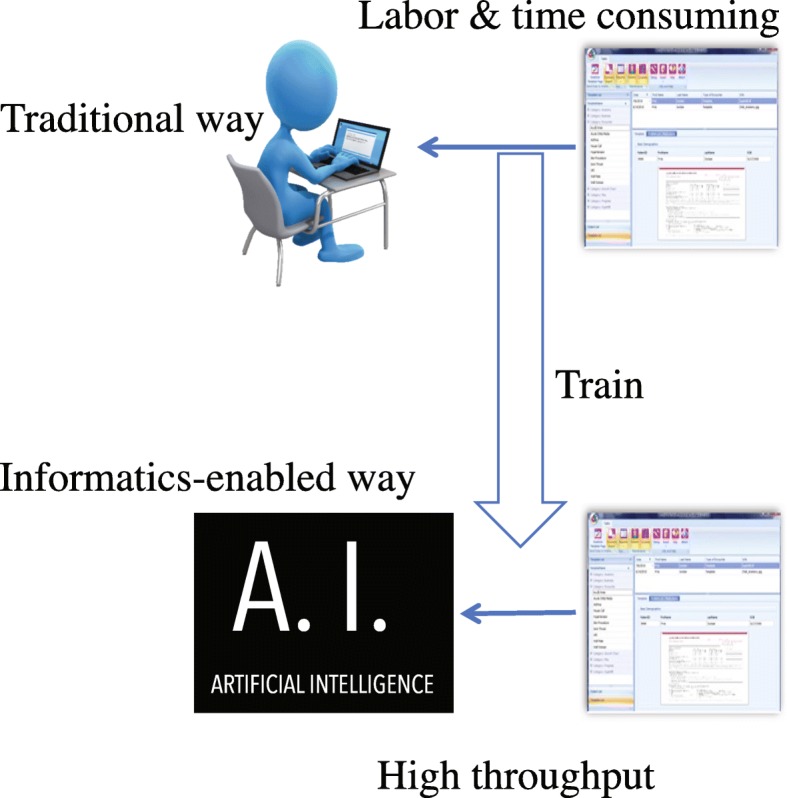
Study rationale

Figure 1 shows the study rationale where the system tries to replicate human abstraction and in turn can help to discover human errors in the “reference standard” of annotated cohort [15]. The current study tried to manually compile rules and algorithms by leveraging a small set of an existing cohort in order to build an automatic high-throughput extraction system for the purpose of accelerating data population.

**Fig. 2:**
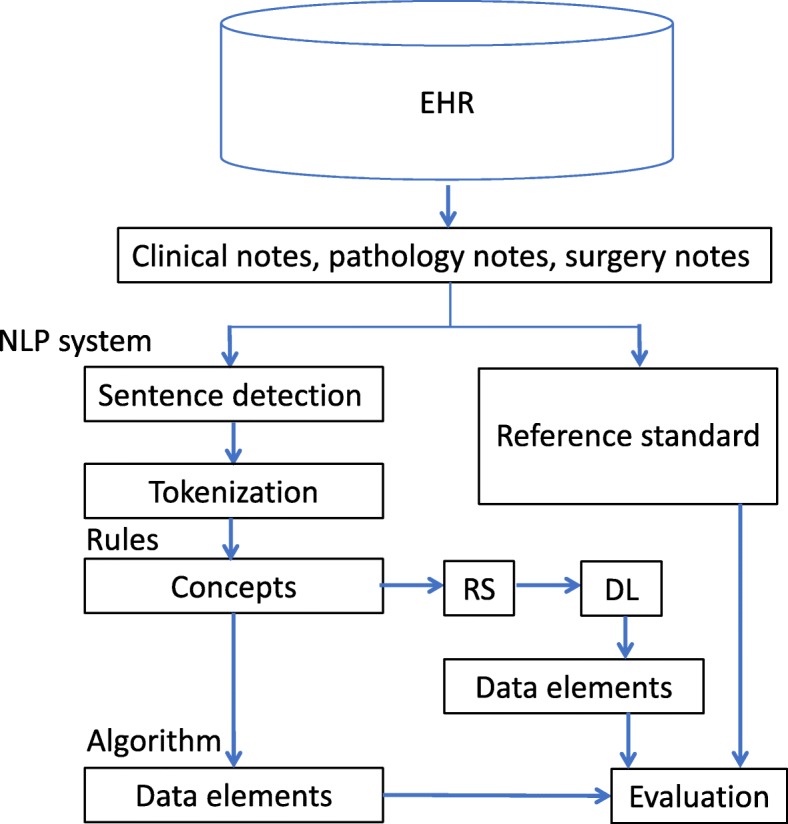
Study design. EHR: Electronic Health Record, RS: related sentences, DL: deep learning

Figure 2 shows the overall study design. Based on an existing lung cancer cohort, an information extraction system was developed using the open source clinical NLP pipeline MedTagger as the platform [16]. Specifically, we utilized the sentence detection and tokenization parts in MedTagger; then, the system integrated rules and algorithms to generate final normalized concept names for each data element, see “Rules and data elements” and “Algorithms” sections for details. We evaluated results of the rule-based NLP system against the human abstracted results on an existing dataset. In addition, to further validate results of the rule-based system, deep learning was used to predict values of data elements using sentences labeled by the rule-based NLP system as input. Finally, we analyzed the rule-base NLP system results and deep learning prediction results against the reference standard in error analysis for histology extraction. The use of deep learning for error analysis intended to introduce a second automation methodology, helping to identify potential error in the reference standard prepared by human abstractors. The following details the data sources and data elements, cohort description, rules and data elements, algorithm, evaluation and word embedding.

### Data sources and data elements

Clinical notes, pathology reports and surgery reports from Mayo Clinic EHR were the primary data sources. An existing lung cancer cohort was used as another data source for rule development and evaluation.

Data elements included stage, histology, tumor grade, chemotherapy, radiotherapy and surgery. Identification of chemotherapy, radiotherapy and surgery was binary, i.e., if the patient was on the therapy or not. While stage, histology and tumor grade are categorical. Table 1 shows the data elements contained in each data source.

**Table 1:** Data elements contained in each data source

Data Elements	Data Sources			
	Clinical Notes	Pathology Reports	Surgery Reports	Existing Dataset
Stage	✔	✔	✔	✔
Histology	✔	✔	✔	✔
Tumor Grade	✔	✔	✔	✔
Chemotherapy	✔	**✕**	✔	✔
Radiotherapy	✔	**✕**	✔	✔
Surgery	✔	**✕**	✔	✔

To replicate results from manual extraction, time window or file types have been limited for pathology reports and surgery reports. Specifically, pathology reports (Cytology Report, General Pathology Report and Consultation Report) between 14 days before and 30, 60 or 90 days after lung cancer diagnosis were used for identifying stage, histology and tumor grade. Surgery reports between 14 days before and 30, 60 or 90 days after lung cancer diagnosis were used for identifying stage, histology and tumor grade, while surgery reports between 14 days before and 365 days after lung cancer diagnosis were used for identifying therapies including chemotherapy, radiotherapy and surgery. All longitudinal clinical notes have been used without any limitation. Clinical notes, pathology reports and surgery reports were processed by the NLP system separately, and then results from each data source were combined for analysis.

### Cohort description

Our study leveraged an existing lung cancer cohort containing 2311 patients definitively diagnosed with primary lung cancer from 2000 to 2012. Previously human abstractors did manual chart review to obtain histological type, tumor stage and grade, and cancer therapies, i.e., chemotherapy, radiotherapy and surgery if available for each patient. In this study, texts from various data sources, i.e., clinical notes, pathology notes and surgery notes were retrieved from Mayo Clinic EHR at Rochester site. Then a corpus of 6737 pathology reports, 135,698 clinical notes, 4781 surgery reports associated with 2307 lung cancer patients from 1999 to 2016 was obtained. We randomly selected 100 lung cancer patients and retrieved associated texts from each data source for corpus analysis to derive language expression patterns. The remaining patients with corresponding data elements were used as reference standards for evaluation of the NLP system.

### Rules and data elements

The findings from randomly selected 100 patients plus expert knowledge were used to define rules for each data element. We iteratively improved the rules on this dataset and then finalized. The rules use regular expression to identify specific concepts for various data elements. For example, to identify histological types of lung cancer in pathology reports, some keywords with same histological types need to be excluded such as “prostate”, “thyroid”, etc. Language patterns in clinical notes are more diverse than pathology reports and surgery reports. For example, “surgery” concept may be mentioned in surgery reports as a specific surgery type as “Segmentectomy”, but may be indicated in clinical notes by “status post lung cancer surgery”. “Stage” was often shown after the word “pathology” or “biopsy” in clinical notes, but usually in the “diagnosis” section of pathology reports. We integrated all patterns into our system. Errors that were tuned during the training process include missing keywords such as “combined modality” that implicates radiation therapy and incorrect sentence splitting.

We used the histological types in the 2015 World Health Organization Classification of Lung Tumors [17] as our keywords to extract histological types. In general, there are two histological types, small cell and non-small cell. Non-small cell includes more subtypes, such as adenocarcinoma and squamous cell. Our rules extract all histological types and then normalize to targeted types in Table 2. Historically Mayo Clinic used different stage and grade criteria for lung cancer.

**Table 2:** Normalized histological types and sub-types in the NLP system

Histological types	Sub-types
Small cell	Small cell
Non-small cell	Adenocarcinoma Squamous Large / larger neuroendocrine Adenosquamous Carcinoid Carcinoid (typical / atypical) Non-small cell (NSCLC unspecified) Other NSCLC Other cell type / Unknown

Historically Mayo Clinic used different staging and grading criteria at different times, which resulted in inconsistent concept mentions. For instance, Mayo Clinic has been using a different tumor grade system from the rest of the world, but pathologists could use either Mayo or the standard system (grade 1–4 or I-IV). This resulted in the condition that grade 1–4 or I-IV, or well differentiated, moderately differentiated, poorly differentiated and undifferentiated have been used for tumor grading. We used all lung cancer stage and grade concepts at Mayo Clinic as keywords and then normalize to targeted stages and grades in Table 3 according to a mapping table generalized through expert knowledge. After NLP extraction based on the rules, exact stage concepts for NSCLC include stage Ia, stage Ib, stage IIa, stage IIb, stage IIIa, stage IIIb, and stage IV. And nonexact stage concepts include early stage and late stage. Stage “Extensive” and “Limited” are for SCLC.

**Table 3:** Normalized stages and tumor grade in the NLP system

Standardized Stages	Standardized Tumor Grades
Ia	Well differentiated
Ib	Moderately differentiated
IIa	Poorly differentiated
IIb	Undifferentiated
IIIa	
IIIb	
IV	
Early stage	
Late stage	
Extensive (SCLC)	
Limited (SCLC)	

### Algorithms

Discordance in recording lung cancer related information is common, even in the same source of EHRs. To resolve such discordance, we used the most frequently extracted concepts as the final concept. If a tie exists, we selected an exact stage over a non-exact stage and a more severe concept over a less severe concept.

### Evaluation

Coverage was calculated first for each single source in this study, where coverage is defined as the number of patients who have related text material in each source, if any, within specified time window. For example, coverage of clinical notes is the number of patients who have clinical notes. Coverage of pathology reports is the number of patients who have document types of “Cytology Report”, “General Pathology Report” and “Consultation Report” between 14 days before and 30, 60 or 90 days after lung cancer diagnosis. Coverage of surgery reports is the number of patients who have surgery reports between 14 days before and 30, 60, 90 or 365 days after lung cancer diagnosis.

For system evaluation, the existing dataset excluding those patients for corpus analysis was used as the reference standard. Results from each data source derived from the NLP system were combined for analysis of recall and precision at patient level. Specifically, if a patient had inconsistent results from clinical notes, pathology reports and surgery reports, the result from pathology report was used. If a patient had inconsistent results from clinical notes and surgery reports, the result from clinical notes was used. Otherwise the result from any single data source was used.

Recall referred to the fraction of patients with the data element identified by the NLP system over the total amount of patients with the data element in the existing cohort. In this study we calculated two precisions, Precision1 and Precision2, where Precision1 refers to the fraction of patients with the true data element identified by the NLP system over the total amount of patients with the data element in the existing cohort; and, Precision2 refers to the fraction of patients with the true data element identified by the NLP system over the total amount of patients with the studied data element identified by the NLP system. The difference between Precision1 and Precision2 is in the denominator. The total amount of patients with the data element in the existing cohort is supposed to be larger than the total amount of patients with the studied data element identified by the NLP system, because the existing cohort includes other data sources such as outside materials which are in PDF format and can not be accessed by NLP.

As mentioned above, there are two general histological types, i.e., small cell and non-small cell. Non-small cell includes more subtypes, such as adenocarcinoma and squamous cell. The reference standard of histological type in the existing dataset provides subtypes as far as possible, when no subtype can be manually extracted the general type subtype (i.e., non-small cell) was provided. Therefore, NLP system extracted subtypes were automatically mapped to non-small cell for evaluation. Namely, if reference standard provided only general type, but NLP identified subtypes, the case is deemed as a true positive.

The existing dataset provides exact stage concepts for NSCLC include stage Ia, stage Ib, stage IIa, stage IIb, stage IIIa, stage IIIb, and stage IV, nonexact stage concepts include early stage and late stage when no exact stage can be found. It also provides stages for SCLS, i.e., “Extensive” and “Limited”. In the evaluation, NLP extracted results having only the exact stage were also assigned with a nonexact stage concept of “early stage” if the exact stage was IA, IB, IIA or IIB, and “late stage” if the exact stage was IIIA, IIIB or IV.

For tumor grade evaluation, we used the exact match between NLP extracted results and the results from the reference dataset as true positive.

A related study has shown the effectiveness of deep learning methods to extract frame semantic information from clinical narratives [18]. In this study, we utilized Convolutional Neural Networks (CNN), a widely adopted deep learning method in error analysis, taking histological cell types as an example. CNN is a feed-forward artificial neural network with layers formed by a convolutional operation followed by a pooling operation [19]. In our implementation, we utilized the typical CNN framework that contains embedding layer, convolution layer, and fully connected layer with a softmax function. The number of filters is 128 and the filter size is 5.

**Fig. 3:**
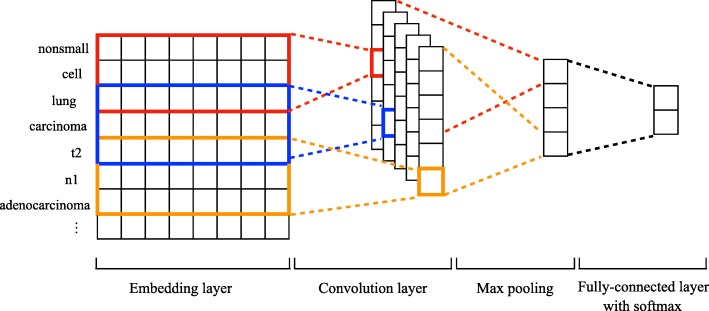
Architecture overview of the CNN model

For the embedding layer, we used a pre-trained word embedding matrix to represent each word in a clinical document in the embedding space. The word embedding matrix was pre-trained by word2vec [20] on a corpus of clinical notes of 113 k patients who received their primary care at Mayo Clinic [21]. Then the sequences for a document from the embedding layer were input to a convolution layer where rectify linear unit (ReLU) was used as convolutional function. 1-max pooling was then performed to choose the most useful feature from each sequence. Finally, to classify the document, we utilized a fully connected layer over global features and a softmax function with the dimension of the number of categories (Fig. 3).

We randomly selected 100 patients as testing set and the remaining patients as training set from the existing cohort for deep learning. Using the classifiers trained from deep learning, we predicted the histological types of the 100 patients using clinical notes, pathology reports and surgery notes.

## Results

Table 4 shows the source coverage for patients. Numbers of patients with pathology reports and surgery reports increased slightly over time.

**Table 4:** Comparison of source coverage

Sources		Coverage
Existing dataset		2311
Clinical notes		2307
Pathology reports	Between 14 days before and 30 days after lung cancer diagnosis	1660
	Between 14 days before and 60 days after lung cancer diagnosis	1835
	Between 14 days before and 90 days after lung cancer diagnosis	1896
Surgery reports	Between 14 days before and 30 days after lung cancer diagnosis	938
	Between 14 days before and 60 days after lung cancer diagnosis	1002
	Between 14 days before and 90 days after lung cancer diagnosis	1023
	Between 14 days before and 365 days after lung cancer diagnosis	1130

Table 5 shows precision and recall for all data elements using the NLP system combining all longitudinal clinical notes, pathology reports and surgery reports of various time windows. The longest time window of 90 days after lung cancer diagnosis provided the best precisions and recalls for histology and tumor grade. Time windows did not affect stage precision and recall appreciably. Precision and recall for chemotherapy, radiotherapy and surgery achieved 100%.

**Table 5:** Precision and recall for all data elements using the NLP system

Data elements	Number of patients in existing Dataset (A)	Number of patients with true NLP results (B)	Number of patients with NLP results (C)	Precision1 (B/A)	Precision2 (B/C)	Recall	Time window
Stage	2127	1330	1883	0.625	0.706	0.885	90 days
	2127	1328	1883	0.624	0.705	0.885	60 days
	2127	1325	1883	0.623	0.704	0.885	30 days
Histology	2208	1918	1989	0.869	0.885	0.982	90 days
	2208	1914	2164	0.867	0.884	0.980	60 days
	2208	1889	2154	0.856	0.877	0.976	30 days
Tumor grade	1635	1182	1203	0.723	0.902	0.801	90 days
	1635	1170	1300	0.716	0.900	0.795	60 days
	1635	1143	1274	0.700	0.897	0.779	30 days
Chemotherapy	1674	1674	1674	1	1	1	365 days
Radiotherapy	769	769	769	1	1	1	365 days
Surgery	312	312	312	1	1	1	365 days

**Fig. 4:**
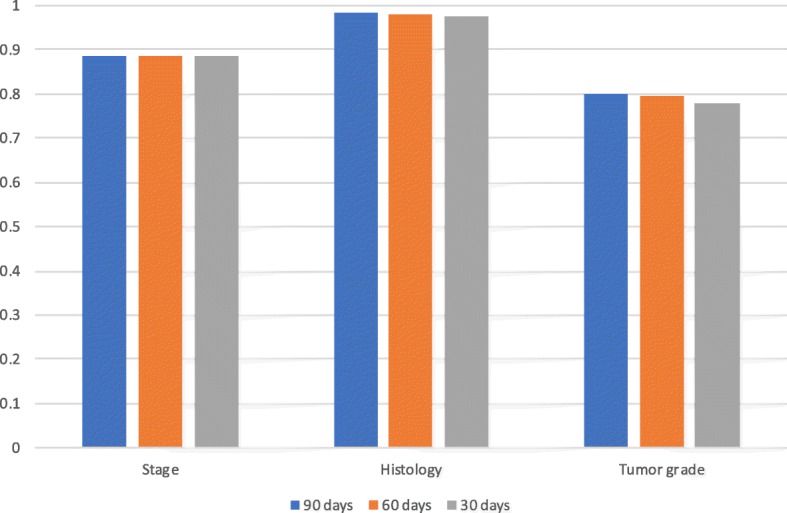
Comparison of recalls using the NLP system combining all longitudinal clinical notes, pathology reports and surgery reports of various time windows. 30, 60 or 90 days refers to using pathology reports and surgery reports between 14 days before and 30, 60 or 90 days after lung cancer diagnosis

Figure 4 shows recalls using NLP system combining all longitudinal clinical notes, pathology reports and surgery reports of various time windows. Recalls for histology across time windows between 14 days before and 30, 60 or 90 days after lung cancer diagnosis were around 98%, recalls for stage around 89%, and recalls for tumor grade ranged from 78 to 80%.

**Fig. 5:**
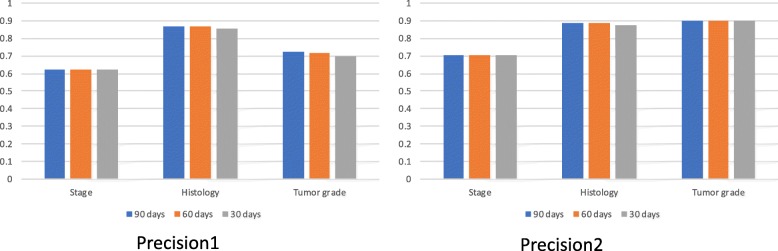
Comparison of precision1 and precision2 using NLP system combining all longitudinal clinical notes, pathology reports and surgery reports of various time windows. 30, 60 or 90 days refers to using pathology reports and surgery reports between 14 days before and 30, 60 or 90 days after lung cancer diagnosis

Figure 5 shows Precision1 and Precision2 using NLP system combining all longitudinal clinical notes, pathology reports and surgery reports of various time windows. Precision2 for histology across time windows between 14 days before and 30, 60 or 90 days after lung cancer diagnosis were around 89%, Precision2 for stage around 90%, and Precision2 for tumor grade around 71%.

Table 6 shows the statistical distribution of each histological cell type in both training and testing data for deep learning. Additional file 1 shows the performance of CNN. We conducted combined analysis following the approach in the evaluation part. Then we analyzed 5 error results that were not in the reference standard but in both the deep learning prediction and the rule-based system extracted results (Table 7). The findings discovered that 4 resulted from the failure of identifying subtypes such as Adenocarcinoma but have been identified as up-level type non-small cell. Further investigation found that 2 patients have no subtype information in EHR including clinical notes, pathology reports or surgery reports. Another 2 patients have related subtype information only in clinical notes, but not in pathology reports, while our algorithm chose the result from pathology reports, i.e., non-small cell, therefore missed the subtype. The Additional 1 patient was labeled as other NSCLC in reference standard, but was identified by both the rule-based system and deep learning as “small cell”. We looked into the EHR, all related data sources including pathology reports, clinical notes and surgery notes indicated the patient had small cell.

**Table 6:** Number of each histological cell type in training and testing data

Histological types	Number (%) in training data set	Number (%) in testing data set
Adenocarcinoma	897 (44.7%)	37 (37%)
Adenosquamous	16 (0.8%)	2 (2%)
Carconoid	1 (0.05%)	0
Carconoid typical /atypical	15 (0.75%)	1 (1%)
Large / larger neuroendocrine	23 (1.1%)	1 (1%)
Non-small cell	342 (17.0%)	15 (15%)
Other cell type /Unknown	1 (0.05%)	0
Other NSCLC	14 (0.70%)	1 (1%)
Small cell	339 (16.9%)	21 (21%)
Squamous	358 (17.8%)	22 (22%)

**Table 7:** Error analysis

Error types	Reason	Number
Failure of identifying subtypes	With no related information	2
	With related information but ignored by algorithm	2
Failure of identifying the type in reference standard	Mistake of reference standard	1

We also looked into the true positives identified by both the rule-based system and deep learning, results showed that we identified more specific histological types, e.g., adenocarcinoma in 8 patients for whom the reference standard provided only up-level type, i.e., non-small cell.

## Discussion

In error analysis, we found that patients with discordance results between reference standard and NLP system and deep learning tend to have less number of clinical notes or related pathology reports and surgery reports, have little related information, or have discordant mentions of data element value. Error analysis findings not only showed the potential to improve the NLP system by optimizing algorithm, but also revealed the areas where NLP system could enhance the reference standard, emphasizing the importance of using automatic methods in improving information extraction for cancer study. Specifically, NLP system helped to identify more specific histological types, e.g., adenocarcinoma in 8 patients that were not provided in the reference standard, and helped to identify correct histology type in 1 patient who was mistakenly identified as another type in the reference standard. The real-world truth is even human annotated data has flaws since human errors are inevitable. There are some limitations in our study. First, stage detection was based on term mentions like “stage IIa”, and we did not extract specific status of tumor, node and metastasis (TNM). In the future study, we will focus on TNM extraction and the development of rules mapping TNM to stage concepts such as “stage IIa”. Secondly, the rules in the NLP system were generated using the EHR from one single institution. Various institutions may use different stage, histology and tumor grade systems from Mayo Clinic. Therefore, the system may not be generalizable to other institutions. However, the NLP part extracting chemotherapy, radiotherapy and surgery may be transferrable to other institutions since these therapy mentions are very explicit in the texts, with identification rate achieved 100% for P and R. Third, the data sources in EHR we used include only clinical notes, pathology reports and surgery reports. Other data sources containing rich information on cancer such as diagnostic imaging reports from CT, MRI and PET need to be studied in the future.

Due to the historic reasons, patient-report error or health providers’ writing error, discordance of recording data elements was very common. In preparing the reference standards, human abstractors often met the same situation where a pathologist would be involved to make the final judgement. In our NLP system, we developed an algorithm to resolve the data discordance issue, where concepts with highest frequency or more advanced concepts have been used. Compared to the previous study on histological type and grade extraction [13], our NLP system obtained the similar precisions (0.88, 0.90). Our study focused on more specific stages such as Ia, Ib, IIa or IIb, not only stage I and stage II. Compared to the previous study on stage extraction [13], our NLP system obtained similar performance in distinguishing more specific stages. The previous study was able to distinguish stage IIIA from stage IIIB with the accuracy in the 64 to 79% range [13]. Our system yielded the precision around 70%. The reason why the performance for stage was not very high maybe because three staging systems have been used in the past 20 years at Mayo Clinic. After all it is challenging even for an expert pathologist to determine the definitive stage. In addition, all performances for therapies were 100%. These findings demonstrated that our NLP rules and algorithms were effective in identifying data elements.

## Conclusion

This study demonstrated the feasibility and accuracy of extracting cancer related information from narrative EHR data for clinical research of lung cancer, as well as the feasibility of improving the efficiency of human abstractors through NLP techniques.

## Supplementary information

**Additional file 1.** Performance of CNN.

## Data Availability

N/A

## Abbreviations

CNN: Convolutional Neural Networks
EHRs: Electronic health records
NLP: Natural language processing
NSCLC: Non-small cell lung cancer
ReLU: Rectify linear unit
SCLC: Small cell lung cancer
TNM: Tumor, node, metastasis
