# Supplementary material for: Natural language processing for populating lung cancer clinical research data
Source: BMC Med Inform Decis Mak. 2019 Dec 5;19(Suppl 5):239. doi: 10.1186/s12911-019-0931-8 (PMC6894100; doi:10.1186/s12911-019-0931-8)
Supplement: Supplementary file 1 — Additional file 1. Performance of CNN. [file 12911_2019_931_MOESM1_ESM.docx]

**Additional File 1. Performance of CNN.**

| Data sources | Precision | Recall | F1-score |
| --- | --- | --- | --- |
| Clinical notes | 0.79 | 0.79 | 0.78 |
| Path reports | 0.60 | 0.70 | 0.63 |
| Surgery reports | 0.28 | 0.42 | 0.33 |
